# Unraveling the secrets of white matter – Bridging the gap between cellular, animal and human imaging studies

**DOI:** 10.1016/j.neuroscience.2014.06.058

**Published:** 2014-09-12

**Authors:** K.B. Walhovd, H. Johansen-Berg, R.T. Káradóttir

**Affiliations:** a Research Group for Lifespan Changes in Brain and Cognition, Department of Psychology, University of Oslo, POB 1094 Blindern, 0317 Oslo, Norway; b Department of Physical Medicine and Rehabilitation, Unit of Neuropsychology, Oslo University Hospital, 0424 Oslo, Norway; c Oxford Centre for Functional MRI of the Brain (FMRIB), Nuffield Department of Clinical Neurosciences, University of Oxford, John Radcliffe Hospital, Headington, Oxford OX3 9DU, United Kingdom; d Wellcome Trust-Medical Research Council Cambridge Stem Cell Institute, John van Geest Centre for Brain Repair, and Department of Veterinary Medicine, University of Cambridge, Cambridge CB2 1QR, United Kingdom

**Keywords:** ASL, Arterial Spin Labeling, CSF, cerebrospinal fluid, DTI, diffusion tensor imaging, DWI, diffusion-weighted imaging, FA, fractional anisotropy, fMRI, functional MRI, MD, mean diffusivity, MRI, magnetic resonance imaging, OPCs, oligodendrocyte precursor cells, PLIC, posterior limb of the internal capsule, ROIs, regions of interest, white matter, myelin, glial cell, DTI, functional imaging, anatomy

## Abstract

The CNS white matter makes up about half of the human brain, and with advances in human imaging it is increasingly becoming clear that changes in the white matter play a major role in shaping human behavior and learning. However, the mechanisms underlying these white matter changes remain poorly understood. Within this special issue of Neuroscience on white matter, recent advances in our knowledge of the function of white matter, from the molecular level to human imaging, are reviewed. Collaboration between fields is essential to understand the function of the white matter, but due to differences in methods and field-specific ‘language’, communication is often hindered. In this review, we try to address this hindrance by introducing the methods and providing a basic background to myelin biology and human imaging as a prelude to the other reviews within this special issue.

## Highlights

• White matter macrostructural measurements explained.
• Introduction to how MRI imaging can be used to understand changes within the white matter.
• Estimation of the white matter microstructures, cells and axons, occupying an imaging voxel.
• Biological methods used for white matter studies explained.

## Introduction

Laid out end to end, the myelinated fibers from one human brain can circle the globe more than three times (Marner et al., 2003). Unraveling the secrets of this remarkable structure raises multiple challenges – not least of which is integrating white matter science at vastly different levels. In myelin biology, myelinated axons are characterized at a nanometer scale and can be imaged at a resolution of cubic micrometers, whereas the measurement units in typical *in vivo* human neuroimaging protocols are on the scale of cubic millimeters, and regions of interest (ROIs) may even span several centimeters. How can these approaches be related to each other? The goal of each approach is ultimately common: to unravel the secrets of white matter and to understand changes that occur in development and plasticity, aging and pathology. How do molecular changes cascade changes that can appear at a macroscale, and how can system-level findings inform myelin biology? Approaches at each scale can help generate testable hypotheses that can be tackled at a cross-disciplinary level. Here we provide a basic outline of the most common methods used within myelin biology and imaging of white matter, to ease integration across fields and to aid reading of the reviews within this special issue.

## White matter: from cellular architecture to human brain imaging

### Part 1

#### What is white matter – the biologist’s view

**Fig. 1:**
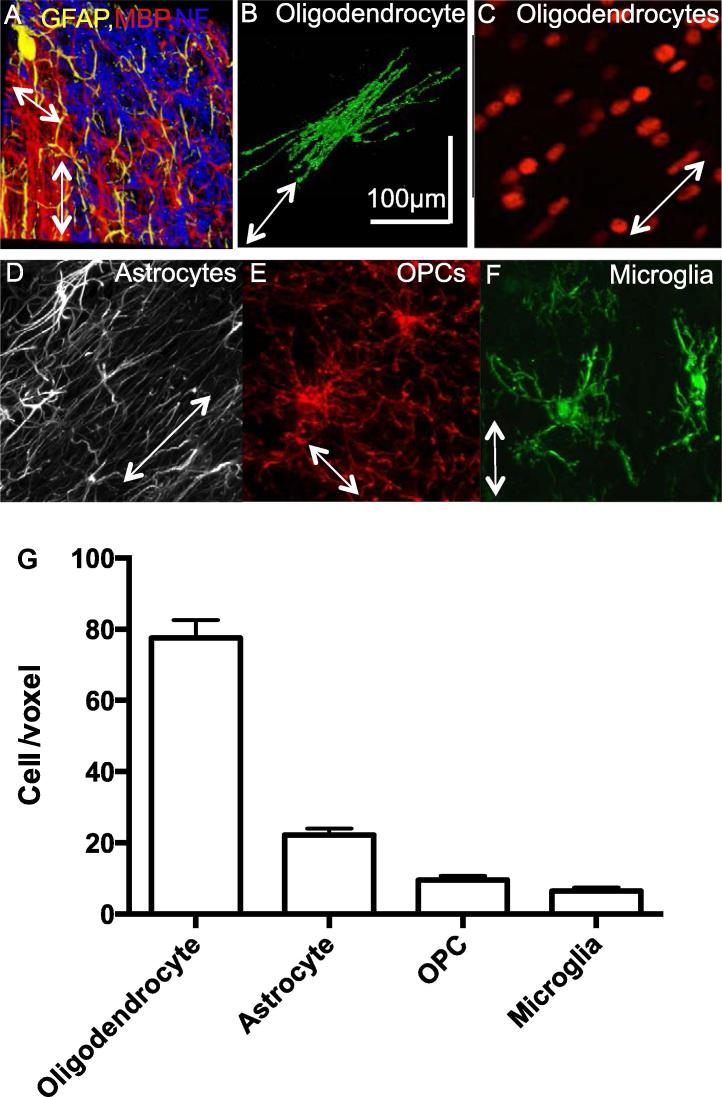
White matter voxel. (A) 100 μm × 100 μm × 50-μm 3D projection of corpus callosum white matter area in adult rodent. Note in this particular area not all the axons are myelinated (yellow: astrocytes; red: myelin; blue: axons). (B) A Lucifer yellow dye filled oligodendrocyte, via patch pipette, in the corpus callosum revealing its morphology and the number of internodes it makes. (C–F) Confocal projections of (C) oligodendrocyte cell nuclei labeled with Olig2 (an oligodendrocyte lineage-specific transcription factor) (D) astrocytes labeled with GFAP, (E) oligodendrocyte precursor cells labeled with NG2 and (F) microglia labeled with Iba1, the length and width of each image is 100 μm × 100 μm and the projection is ∼50 μm deep. White arrows show the direction of the axons. (F) Quantification of the glial cell number (oligodendrocyte identified as Olig2postive cells, astrocytes as GFAP positive, OPCs as NG2-positive cells and microglia as Iba1) in a voxel of 100 μm × 100 μm × 100 μm, from adult rodent white matter tracts (corpus callosum and cerebellum).

At a macrostructural level the CNS white matter is often simplistically conceptualized as a tightly packed tissue with myelinated axonal fibers devoid of neuronal cell bodies (but see Concha, 2014). At a cellular level, the white matter is categorized into myelinated and unmyelinated axons and glial cells of which there are the myelin-producing oligodendrocytes, astrocytes, microglia, and oligodendrocyte progenitor cells (Fig. 1A–F).

During development, at the time most axons have found their targets, oligodendrocyte precursor cells (OPCs) migrate throughout the brain, often along axons (Small et al., 1987, Kakita et al., 2003), and differentiate into myelinating oligodendrocytes, as discussed by Mitew et al., in this issue (Mitew et al., 2014). This sequence of events is orchestrated by a complex array of signaling mechanisms, as reviewed in this issue (Almeida and Lyons, 2014, Mitew et al., 2014, Wang and Young, 2014). After ‘developmental’ myelination is complete some OPCs remain in the adult and constitute ∼9% of all cells in the white matter (Dawson et al., 2003). Theories on the function of these adult OPCs remain speculative (but see Wang & Young, 2014). The other main glial cells in the white matter are astrocytes, which are numerous and with extensively long processes. Particularly, human astrocytes are much longer and more complex compared to the rodent ones (see review in this issue by Lundgaard et al.).

**Fig. 2:**
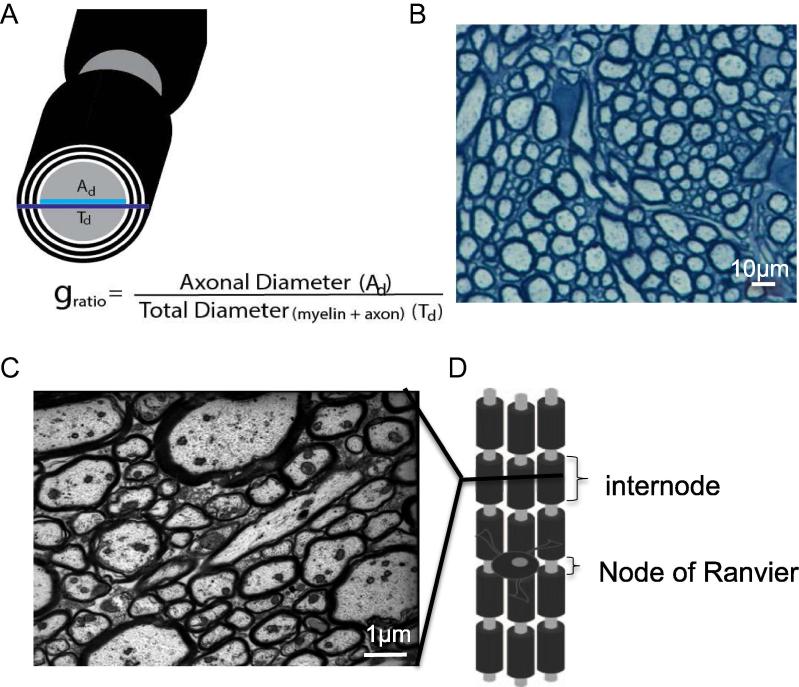
The structure of myelin. (A) A schematic diagram of myelinated axon illustration how *g*-ratio is measured, a parameter used to quantify the thickness of myelin on electronmicrographs as shown in C. (B) Semi-thin section of a cerebellar penduncle, stained with toluidine blue (image is provided by Dr. Helene Gautier). (C) Electro micrograph of corpus callosum (courtesy of Mr. Ginez Gonzalez and Dr. Mark Kotter) showing the variety of axonal diameter and myelin thickness. (D) A schematic diagram of myelinated fibers, illustrating an internode (a myelinated segment along an axon) and the node of Ranvier (the gap between internodes).

Not all axons in white matter tracts are myelinated, in fact very few white matter tracts in the CNS are fully myelinated. There is a great degree of variability between tracts in the number of myelinated axons. Moreover, the proportion of axons that are myelinated along some white tracts, such as the corpus callosum, is greatly variable and area dependent, for example the proportion of myelinated axons in the posterior corpus callosum is much greater than for the anterior corpus callosum. For example some areas of the corpus callosum are only 30% myelinated (Sturrock, 1980). Conventionally, myelin thickness and internodal length (i.e. the length of each myelin segment – the distance between nodes of Ranvier; see Fig. 2D) scale linearly with axonal diameter, as measured by the ‘*g*-ratio’ (see method section and Fig. 2A). However, there is a great variability in the *g*-ratio indicating a range in myelin thickness and internodal length, both between axons and now recently demonstrated also along an individual axons (Tomassy et al., 2014), indicating that myelin thickness and internodal length may be regulated in spatially specific ways. The most plausible and attractive hypothesis is that these parameters are adjusted along axons to regulate axonal conductivity and synchronize the neuronal network (see Pajevic et al. and Seidl 2014). Whether changes in neuronal activity affect the thickness of myelin or internodal length is still uncertain, but it is known that OPCs can sense (Káradóttir et al., 2005, Káradóttir et al., 2008, Kukley et al., 2007, Ziskin et al., 2007) and respond to changes in neuronal activity (Gibson et al., 2014) and that myelination is, at least in part, regulated by neuronal activity (Demerens et al., 1996, Stevens et al., 2002, Lundgaard et al., 2013).

#### Toward a multiscale understanding: From a myelin biology point of view, what does a voxel contain?

To aid integration of information across scales, it is helpful to consider the likely constitution of a typical neuroimaging voxel in cellular terms. To directly address this question we quantified the number of cells and axons in a volume of white matter (100 μm × 100 μm × 100 μm), which mimics the volume of a voxel that is used in high-resolution diffusion magnetic resonance imaging (MRI) scans of rodent brains. We performed this quantification by taking confocal images of brains sections that have been labeled with fluorescent antibodies that target cell-specific proteins (Fig. 1A, C–F) and electron micrograph images (Fig. 2C) from rodent white matter. Extrapolating these numbers to a voxel size of e.g. 2 mm × 2 mm × 2 mm, conventionally used in diffusion imaging in humans, is almost impossible as neuronal size and density do not scale linearly according to brain volume (Herculano-Houzel, 2011) and little is known about how the white matter scales between species.

From electron micrographs of different rodent white matter tracts (corpus callosum and cerebellar peduncle) we quantified the number of axons. The number of axons varied greatly across white matter tracts and within a white matter tract. The range was from 1000 axons per voxel to over 13,000, with a median of 3000 axons. Thus it is difficult to conceptualize how changes in a few neurons will affect changes in measurements from an imaging voxel, especially in a voxel containing >13,000 axons. It becomes even harder to conceptualize with increasing voxel size, assuming equal density of axons in human white matter to rodent white matter the 2 mm × 2 mm × 2-mm voxel would contain from ∼0.5 million axons to >5 million.

However, we found that glial cell numbers varied less than axonal number both within and between tracts. Oligodendrocytes were found to be the most abundant glial cells in the white matter, with average counts of 86 ± 9 cells/voxel (Fig. 1C, G). Astrocytes were found to be the second most abundant, at 22 ± 2 cells/voxel. Although astrocytes were four times fewer than oligodendrocytes (Fig. 1D, G) their processes covered a large area, amounting to ∼48% of a voxel, which covers a similar volume as myelin (also ∼48%). Thus it is important to acknowledge that astrocytes can make a significant contribution to white matter signals arising from a voxel (100 μm × 100 μm × 100 μm). Assuming unchanged cell size and density between the rodent and the human white matter we estimate ∼700,000 oligodendrocytes and ∼180,000 astrocytes being present in a 2 mm × 2 mm × 2-mm voxel in a human imaging study. However, human astrocytes are 2.5 times bigger and occupy 16.5-fold greater volume than rodent astrocytes (Herculano-Houzel, 2014). Thus, if astrocyte cell number remains the same between human and a rodent white matter (which is unlikely), astrocytes will generate more impact on signals generated from a voxel in a human study than rodent study. However, whether astrocyte cell numbers scale down with their increased size between human and rodent is currently unknown. Microglia and OPCs were found in similar numbers in the white matter. In a voxel of 100 μm × 100 μm × 100 μm we counted 6.5 ± 1 OPC and 9.5 ± 1 microglia (Fig. 1E–G). This numbers of OPCs and microglia are highly consistent with their reported cellular density in the rodent white matter (Dawson et al., 2003). Due to lack of knowledge on how OPCs and microglia scale across species, assuming the size and density of these cells for the human brain to be equal to the rat, results in estimates of 52,000 OPCs and 76,000 microglia in a 2 mm × 2 mm × 2-mm voxel.

In the white matter most cells have their processes roughly aligned along with axons, on the contrary to the gray matter where the processes are more omnidirectional. It is thus conceivable that the cell’s processes, given that they are both numerous and longer than the cell body, play a more important role in the signals generated per voxel during imaging than the cell body. How much microglia and OPCs influence the signals per voxel is debatable, given their low number and relative short processes. Moreover, it is questionable whether any changes in their number or process alignment will have any significant influence on signals measured from a voxel. However, from our quantification the major player in signal induction, apart from oligodendrocytes, myelin structure and axons, are undoubtedly astrocytes, with their long processes that contribute to a large amount of the voxel ∼48%. Thus, changes in astrocyte number, process alignment or thickness could have a significant influence on measurements taken from a single voxel, especially in humans where these cells can have extremely long processes that reach up to 1 mm long (Oberheim et al., 2009).

#### Introduction to methods for studying white matter

##### Microscopic imaging of white matter

The following sections aim to provide the reader with basic background to methods used to study white matter, ranging from cellular techniques to human neuroimaging approaches.

##### Light microscope imaging to study the cells in the white matter: 12–250-μm-thick brain sections

Immunohistochemistry utilizes a ‘primary’ antibody that is designed and made against a cell-specific epitope (most commonly a section of a protein), which it binds strongly to. This is then visualized by utilizing a secondary antibody conjugated to a fluorophore (as can be seen in Fig. 1) or other molecules such as enzymes, for example horseradish peroxidase, that allow for more permanent visualization of proteins when a substrate (such as DAB) is added. This method is extensively used in cell biology to identify cells and their differentiational stage and expression of specific functional proteins. These can range from nuclear markers, such as marking proteins needed for transcription of specific site of the genome and even labeling chromatin status, to cytoplastic proteins and surface markers. This method can allow for a mechanistic insight into cell biology as well revealing cell morphology and enabling cell identification, as specific cells express cell-specific proteins.

##### 1–10-μm-thick sections or semi-thin brain sections – to study white matter structure and myelinated fibers

Myelinated axons can be readily visualized on semi-thin gluteraldehyde-fixed brain samples when the sections are cut thin and labeled with myelin stains (or membrane dyes), such as toluidine blue or luxol fast blue. In these sections myelin is stained dark whereas neurons take up less colored dye (Fig. 2B). This method is useful to get information on white matter ‘health status’, and is therefore extensively used in remyelination and white matter disease studies.

##### Electron microscope studies of white matter, ultrastructure of myelin and axons: <100-nm-thick sections

PFA or gluteraldehyde-fixed and osmium post-fixed resin-imbedded brain samples are cut into ultrathin (<100 nm) slices and then processed for transmission electron microscopy. Myelin (due to the osmium which serves as both a fixative and a stain as it binds to lipids) is visualized as dark rings around the axons (Fig. 2C). Electron microscopy is currently the only method with high-enough resolution to allow for detailed quantification of white matter structure, such as number of myelinated axons, thickness of myelin and myelin ultrastructure. The most common parameter used to identify changes in myelination is the *g*-ratio of myelinated axons (Fig. 2A). The number of myelin lamina (thus myelin thickness) increases with axonal diameter, in an almost linear fashion for axons below 5 μm, thus by using the *g*-ratio, i.e. normalizing to axonal diameter, the thickness of myelin can be compared between axons, tracts and subjects. Unmyelinated axons will have a *g*-ratio of 1, thus the lower the *g*-ratio the thicker the myelin. The optimal *g*-ratio for the CNS has been estimated to be ∼0.77 (Chomiak and Hu, 2009) quite close to the actual mean *g*-ratio of myelinated axons in the optic nerve (Chau et al., 2000).

#### The future at the nanoscale

Recent advantages in electron microscopy have made it possible to visualize myelinated axons on frozen tissue with incredible detail and clarity (Snaidero et al., 2014) providing details of the mechanisms of myelination. This along with conveyor belt sectioning of brain samples allowing for individual axons to be reconstructed ultrastructurally with a voxel resolution of 30 × 30 × 240 nm, revealed that along a single axon myelin segment length varies with often large sections being bare (Tomassy et al., 2014). Along with advances in super-resolution light-microscopy, which now provides unprecedented opportunities to image myelination in action and to detect white matter changes at a 1-μm^3^ voxel resolution *in vivo*, these novel methodologies will revolutionize our understanding of white matter.

#### Methods for imaging human white matter *in vivo*

White matter can be imaged in living human brains using MRI. MRI utilizes strong magnetic fields and pulse sequences and typically relies on radio frequency signals emitted by excited hydrogen atoms in the brain tissue, which contain varying extents of water molecules. Contrast between brain tissue types is determined by the rate at which the excited atoms return to their equilibrium states.

##### Imaging CNS anatomical features

**Fig. 3:**
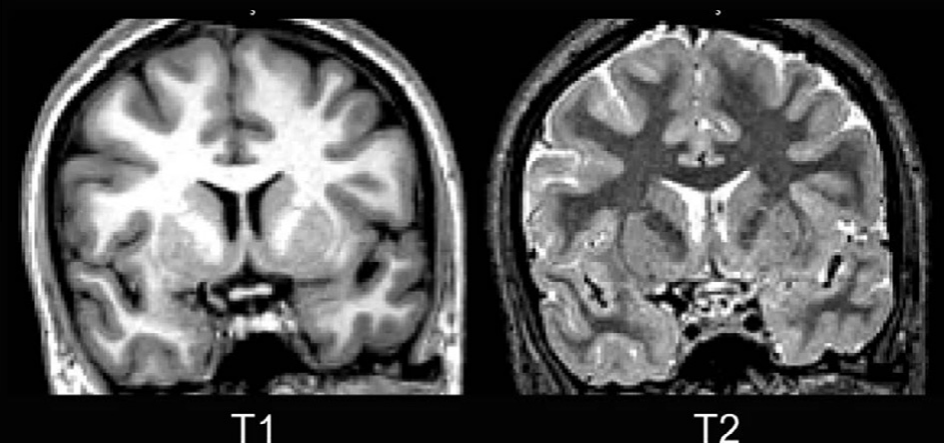
T1- and T2-weighted brain scans. Here, two coronal images from the same young female (age 20 years) are shown in the coronal view. As can be seen, they show inverse intensities. In the T1-weighted image (left panel), compartments with greater water contents appear darker, so that cerebrospinal fluid (CSF) has the darkest intensity, gray matter (for instance in the cortical ribbon, basal ganglia and hippocampi) intermediate intensities, while white matter shows brighter intensities. Such images are often used for reconstruction and quantitative analyses of subcortical and cortical structures. The T2-weighted image (right panel) show inverse intensities with white matter appearing darker, and may be useful for visualizing features such as white matter lesions. When using both types of scans in combination, and T1/T2 ratios, contrast can be improved, and this can be used for myelin mapping (see also Fig. 7).

**Fig. 4:**
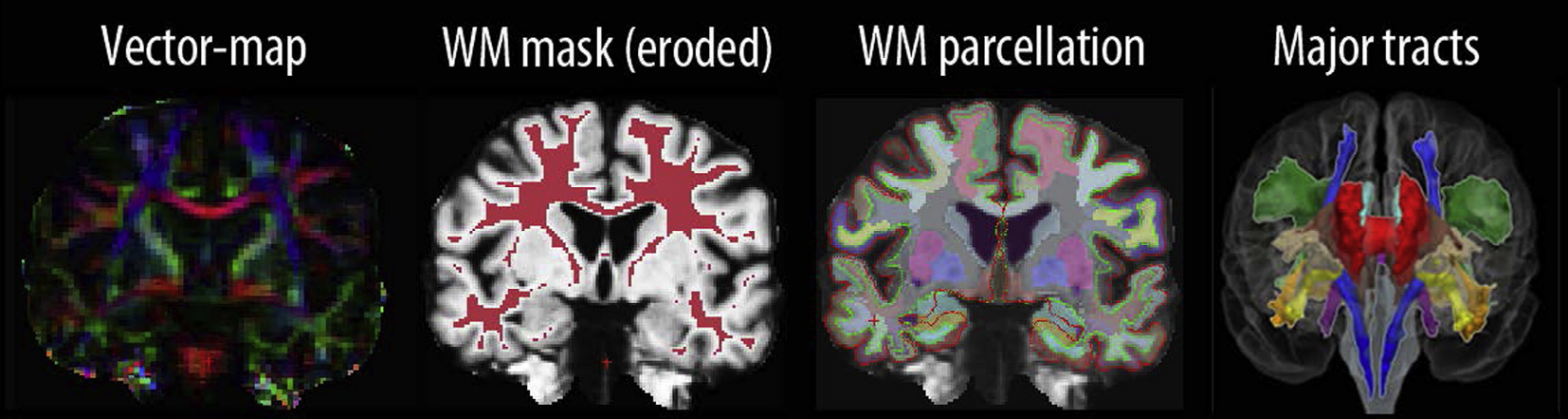
Multiple examples of ways of measuring and reconstructing white matter properties based on MRI scans.

For visualizing brain macrostructure, scan parameters can be varied to show different anatomical features. *T1-weighted images* are often used for analyses of brain macrostructure in neuroscience. They depict the spin–lattice (T1) or longitudinal relaxation times of different types of brain tissues, producing good soft tissue contrast. Essentially, compartments with greater water content will appear darker, whereas compartments with increased lipid content (like myelinated white matter structures) will appear bright. Please see Fig. 3 for a scan showing good contrast between cerebrospinal fluid (CSF), white matter and gray matter. These images can be reconstructed and quantified in different ways to yield submillimeter measures of subcortical and cortical structures (Fischl et al., 2002, Reuter et al., 2012), see Fig. 4. Such a level of precision has been validated by histological analyses (Rosas et al., 2002). *T2-weighted images* offer a different type of contrast (Fig. 3, reflecting spin–spin, or transverse relaxation; where regions with high water content appear bright), and are particularly useful for delineation of ventricles and for visualization of brain pathology, such as white matter lesions. While different scan types are typically used for certain purposes, however, the delineation of white and gray matter in differently weighted images will also depend on the maturational state of the nervous system, as discussed by Dubois et al. in this issue (Dubois et al., 2014). Different pulse sequences result in intensity contrast that primarily reflects other features, such as proton density of the tissue (PD-weighted scans). In addition, specific tissue types (like fat or CSF) may be suppressed to improve visualization of otherwise obscured structures. Various scans may also be combined to improve signal quality (Jog et al., 2013) and analysis possibilities, to be further discussed below.

##### Imaging CNS white matter microstructure – diffusion MRI

**Fig. 5:**
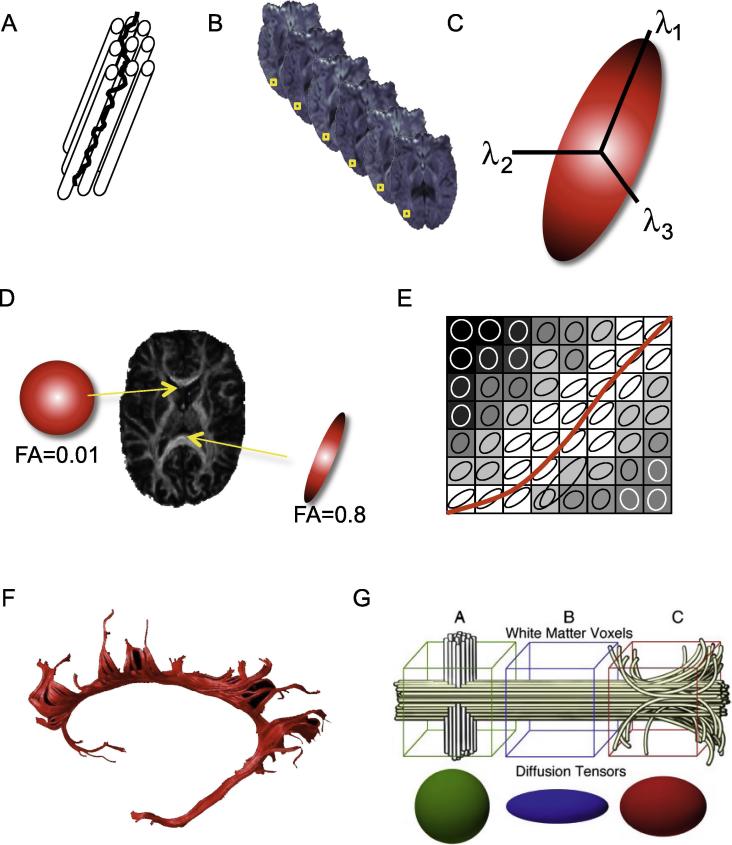
Contributions to tract anisotropy. (A) Water diffuses more easily along the axis of a fiber bundle than it does across the axis of the bundle, due to the presence of barriers such as membranes and myelin. (B) Typically, multiple different diffusion-weighted images are acquired, with each one sensitized to diffusion along a different direction in space. (C) One can fit a mathematical model to the measurements in order to estimate certain model parameters that describe diffusion behavior within each voxel. The most commonly used model, the diffusion tensor model, fits the measurements to a tensor, or ellipsoid, which is fully characterized by its three orthogonal eigenvectors and their associated lengths, or eigenvalues (*λ*_1_, *λ*_2_, *λ*_3_). (D) In cerebral spinal fluid (CSF), water diffuses freely in all directions and so FA is close to zero; in white matter, diffusion is directionally dependent and so FA is closer to one. (E) The long axis of the diffusion tensor corresponds to the principal diffusion direction. Within a coherent fiber bundle this aligns with the fiber direction. (F) By following these voxel-wise estimates of principal diffusion directions it is possible to perform diffusion tractography, and reconstruct estimates of fiber pathways. (G) Variations in diffusion parameters along tracts during normative development are likely a combination of tract-specific (e.g. myelin content, axonal characteristics) and local environment contributions. Voxel 1 contains a tract of interest (yellow) as well as a crossing tract (gray), resulting in low anisotropy measurements at this point. Voxel 2 contains only the tract of interest and exhibits high anisotropy. Within voxel 3 axons from nearby gray matter join the tract and some axons break off heading toward gray matter targets. The result would be a drop in anisotropy measurements at this point in the tract. The figure is from (Johnson et al., 2013).

Diffusion-weighted imaging (DWI) was invented in the mid-eighties and continues to be increasingly successfully developed (Le Bihan et al., 1986). DWI maps the diffusion of water molecules. While free diffusion of water is equal in all directions, i.e. isotropic, this is to varying degrees not the case in the central nervous system tissue, where diffusion gets restricted by fibers, membranes and so forth (Fig. 5A). If these restricting structures are directionally oriented then this creates a directional dependence, or ‘anisotropy’, in water diffusion, which can then reveal microstructural properties of the brain.

In diffusion MRI we use spatially varying gradients to measure water diffusion along multiple different directions in space (at least 6, often many more) (Fig. 5B). We can fit a mathematical model to those measurements at each voxel, in order to estimate certain useful model parameters. The most commonly used model, the diffusion tensor model, fits the measurements to a tensor, or ellipsoid, which is fully characterized by its three orthogonal eigenvectors and their associated lengths, or eigenvalues (*λ*_1_, *λ*_2_, *λ*_3_) (Fig. 5C) (Basser et al., 1994). The size and shape of the ellipsoid tells us about the amount and directional dependence of diffusion at that voxel. Mean diffusivity (MD) is the average of the three eigenvalues and quantifies the overall amount of diffusion in the voxel. Fractional anisotropy (FA) is a parameter that characterizes the shape of tensor by comparing the different eigenvalues, and ranges from zero (spherical) to one. In CSF, water diffuses freely in all directions and so FA is close to zero; in white matter, diffusion is directionally dependent and so FA is closer to one (Fig. 5D–E).

Differences in FA are found with development and aging, in clinical groups, and in relation to behavioral variation in healthy people, as reviewed elsewhere (Johansen-Berg, 2010) and in this issue (Bennett and Madden, 2014, Dubois et al., 2014, Salat, 2014, Treit et al., 2014, Concha, 2014, Amlien and Fjell, 2014, Paus et al., 2014). It is therefore thought to reflect tissue characteristics that have functional relevance. Indeed, physical properties of the fiber bundles, such as packing density, myelination and axon diameter are known to influence FA (Beaulieu, 2009). However, faced with a difference in FA between two populations, it is not possible to be sure which structural property of the white matter underlies the difference without additional information (Zatorre et al., 2012). In tightly controlled model system, modulating either axon integrity or myelin can be shown to impact specifically on axial (*λ*_1_) or radial (*λ*_2_ + *λ*_3_/2) diffusivity (Song et al., 2003). It can therefore be tempting to interpret these parameters as specific measures in human studies, but this should be discouraged due to the presence of complex fiber architecture and orientation uncertainty (Wheeler-Kingshott and Cercignani, 2009). Although diffusion tensor imaging (DTI) parameters such as FA or diffusivities are sensitive to tissue microstructure, there is not a one-to-one relationship between these parameters and a particular physical property such as myelination or axon diameter. Researchers are attempting to develop more sophisticated biophysical models of diffusion data, in which model parameters are tissue properties, such as axon diameter (Assaf et al., 2008, Alexander et al., 2010). Such models would allow for more definite biological interpretations to be made.

Another major application of diffusion MRI is for reconstruction or fiber pathways, or ‘tractography’. The long axis of the diffusion tensor corresponds to principal diffusion direction. Within a coherent fiber bundle this aligns with the fiber direction (Fig. 5F). By following these voxel-wise estimates of principal diffusion directions it is possible to perform diffusion tractography, and reconstruct estimates of fiber pathways (Fig. 5G) (Jones et al., 1999).

While the diffusion tensor model can be a useful simple approach for characterizing coherent fiber bundles, this model does not adequately describe areas of fiber crossing of complexity. High-angular resolution diffusion imaging (HARDI) involves acquiring more images, and so sampling more diffusion directions. This allows for more complex models to be fit to the data, enabling estimation of multiple fiber orientations within each voxel. The key advantage to fitting multiple fibers to each voxel, is that it is then possible to track crossing fibers. One class of multi-fiber models uses probabilistic approaches to estimate the uncertainty associated with each orientation (Parker and Alexander, 2005, Behrens et al., 2007). In addition to sampling different diffusion directions, it is also possible to sample different water populations, with different degrees of restricted diffusion. One class of such schemes is called diffusion spectrum imaging (DSI) (Wedeen et al., 2005). One extension of this scheme, ‘restriction spectrum imaging’ (RSI), provides information on both orientation and length scale of water diffusion (White et al., 2013). This is argued to allow for separate estimation of diffusion from extraneurite and intraneurite water, potentially providing new insights into complex fine-grained tissue microstructure.

##### Imaging based on blood flow: perfusion and functional MRI (fMRI)

White matter is less perfused than gray matter, and hence many perfusion and fMRI studies, which also rely on blood flow, focus on gray matter. However, as perfusion and fMRI may also be used in studies of white matter, we include a short description here. Measures of cerebral perfusion describe passage of blood through the brain’s vascular network, and depend on serial measurement of concentration of a tracer agent in the brain (Petrella and Provenzale, 2000). Exogenous tracers such as paramagnetic contrast material have been used, but also an endogenous and hence non-invasive tracer, namely magnetically labeled blood, as in Arterial Spin Labeling (ASL), is applied. ASL was until recently considered unsuitable to measure stable white matter blood flow, but technical advances now make this more feasible. Relatively long scan times can be required and methodological complications remain, but ASL is a potential *in vivo* microvascular parameter to investigate white matter changes (van Osch et al., 2009, Mutsaerts et al., 2013), e.g. white matter lesions can show altered perfusion. Regardless of the challenges of measuring perfusion in white matter per se it should be noted, as discussed by Salat in this issue (Salat, 2014), that cortical blood flow can be strongly associated with white matter integrity as measured otherwise, by DTI (see below) (Chen et al., 2013).

Recent data also indicate that blood oxygen level-dependent (BOLD) contrast, as typically used for fMRI, may be used to study aging and pathology effects within white matter (Makedonov et al., 2013). In recent years, there has been much focus in fMRI studies on what is often termed “functional connectivity”. These studies, however, assume connectivity based on correlations among activity patterns across brain areas, and not white matter tissue per se. Such measures of functional connectivity have to some extent been shown to relate to white matter structural connectivity as indexed by structural (Zito et al., 2014) and diffusion-weighted imaging (van Oort et al., 2013). Such relationships in aging are further discussed by Bennett and Madden in this issue (Bennett and Madden, 2014).

### Part 2

#### White matter – the multimodal imaging perspective

The methods described above have been used to study changes in white matter with learning, development and aging.

#### Plasticity

DTI studies of healthy adults have convincingly shown that training of e.g. juggling (Scholz et al., 2009), working memory (Takeuchi et al., 2010), and episodic memory (Engvig et al., 2012) are accompanied by regional increases in white matter FA. However, decrease of FA has also been shown in relation to increasing balancing skills (Taubert et al., 2010). Increases in FA have been interpreted as possibly related to growth of axons or increased myelination, while decreases may be related to increased e.g. fiber crossing/changes in axonal diameter. However, a small number of animal models on training combining DTI and histological/immunohistochemical analyses now exist to hint at the underlying mechanisms (Blumenfeld-Katzir et al., 2011, Sampaio-Baptista et al., 2013).

## White matter lifespan changes

Enormous brain changes take place throughout the lifespan. Volumetric growth is dramatic in infancy, with the brain nearly tripling its size from birth to two years of age (Dekaban, 1978). While nearly all neurons are in place at birth, myelination is most prevalent postnatally. Still, as observed by structural MRI, brain growth is dominated by gray matter increases in the first year of life, and a slower increase thereafter (Gilmore et al., 2012), followed by largely monotonous decreases of gray matter in school age, adolescence and throughout adult life (Østby et al., 2009, Walhovd et al., 2011). For white matter, however, a very different lifespan trajectory is observed by imaging studies: White matter volume has been found to increase throughout childhood, adolescence (Østby et al., 2009), and well into adulthood (Westlye et al., 2010, Walhovd et al., 2011). Volumetric imaging studies typically find that white matter volume peaks for the cerebrum as a whole in the forties or fifties, and accelerating decline in white matter volume is observed starting in the sixties (Westlye et al., 2010, Walhovd et al., 2011).

**Fig. 6:**
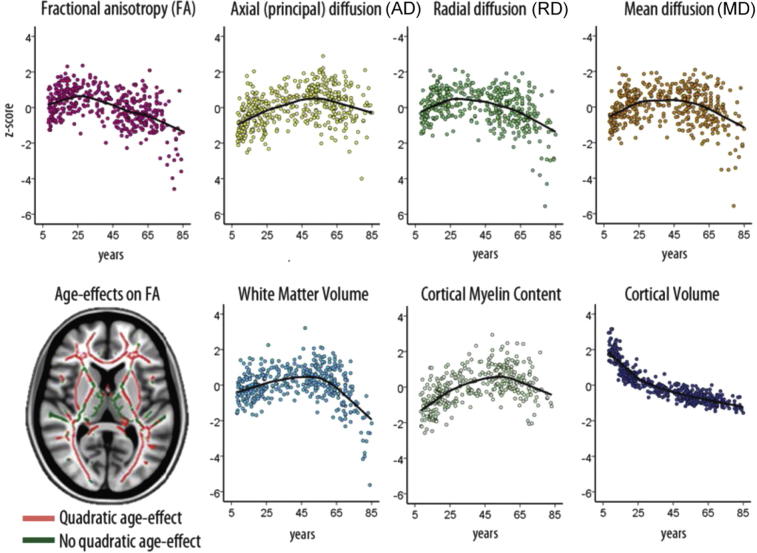
Multimodal imaging of white matter through the lifespan. Results are based on 430 well-screened healthy participants between 8 and 85 years (mean 41.6 years). Values in the scatterplots are expressed in *z*-scores (standard deviations) to ease comparison between metrics. Values represent for FA, axial, radial and mean diffusion the mean of all voxels that were included in the left superior longitudinal fasciculus. The tract-based spatial statistics skeleton represents the middle of the tract for all participants (red and green voxels in the lower left brain image). White matter volume represents the total volume of all cerebral white matter, and cortical volume represents the volume of all cortical gray matter, in both cases corrected for total intracranial volume. Cortical myelin content is based on the ratio between T1- and T2-weighted MR images in an overlapping sample (*n* = 339, age 8–83 years), sampled 0.2 mm from the white matter/gray matter boundary into the gray matter in the superior frontal cortex.

Hence, and as shown in Fig. 6, multimodal imaging can be used to delineate the dynamic interplay between white and gray matter changes in the brain. However, the cellular foundation of the observed changes remains poorly understood. For example, as noted by Sadeghi et al. (2013), FA of the splenium and the posterior limb of the internal capsule (PLIC) are approximately the same values at birth, yet in contrast to PLIC, the splenium is not myelinated at that stage. Differences in RD of these regions are observed, and hence the high FA value of the splenium may be due to its high density of axons (Sadeghi et al., 2013). As noted by Concha in this issue (Concha, 2014), the interpretation of diffusion parameters of white matter rests on knowledge of what is known to drive diffusion anisotropy, namely axonal membranes, density and coherence, as well as myelin sheaths. Such knowledge is starting to accumulate from animal models, but these factors interact to modulate anisotropy, and as emphasized by Concha, animal models may not necessarily provide accurate representation of the human condition (Concha, 2014). Histological confirmation is crucial to understand imaging parameters not only in neurological conditions, but also in normal development and aging. It has been estimated, based on careful histological analyses, that the young adult human male brain contains 176,000 km of myelinated fibers, but the cross-sectionally estimated age-loss is about 45% from the age of 20 to 80 (Marner et al., 2003). We now need to couple the DTI findings and also macrostructural and contrast parameters in human beings, where widely different processes can cause apparently similar imaging effects (see Fig. 5), to findings from cellular biology and histology.

**Fig. 7:**
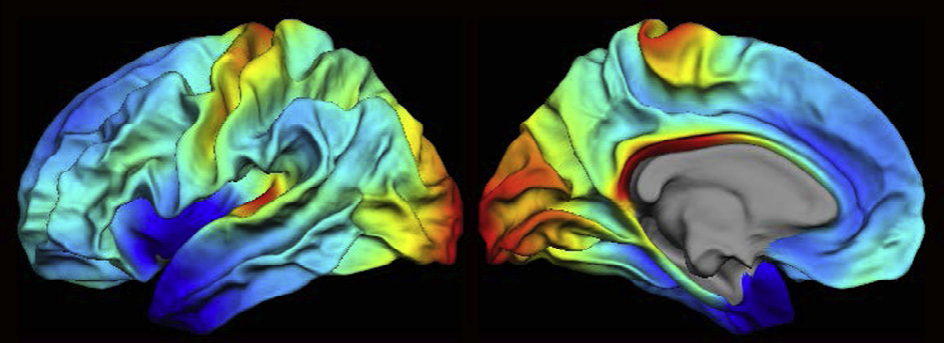
MRI myelin mapping. T1w/T2w ratio myelin maps from a group of young subjects (*n* = 85, 43 females (50.6%), mean age (SD) = 14.7 (3.3), min–max age = 8.4–19.7). Data are shown for the left hemisphere (left panel: lateral view, right panel: medial view). Values were sampled across cortex (20 times, at a 5% spacing) along the normal from the pial to the white surface, and then averaged. The T1w/T2w ratios are dimensionless quantities, and values are displayed between third and 96th percentiles with saturation below (black) and above (white) these values to allow for comparisons of data from different imaging acquisition parameters; for instance, the myelin maps show striking similarities with the original maps presented in Glasser and Van Essen (2011). Figure by Håkon Grydeland.

A troublesome example of prevailing uncertainty regarding neurobiological foundations, is the observed cortical thinning ongoing for most of the lifespan, in school-age children, adolescence, young and older adults (Tamnes et al., 2010, Brown and Jernigan, 2012, Brown et al., 2012). Early on, this cortical thinning must represent maturational processes, likely including pruning of synapses (Huttenlocher et al., 1982), but perhaps most predominantly intracortical myelination, accompanied by cognitive gains. Later in adulthood, however, cortical thinning represents mainly negative changes, including neuronal shrinkage, loss of synapses, dendritic spines, and – in neurodegenerative disorders – marked neuronal loss, all accompanied by cognitive decline (see Fig. 6). Using a combination of T1- and T2-weighted myelin mapping and diffusion tensor imaging (see Fig. 7), Grydeland et al. found that intracortical myelination was ongoing until the late 30s, followed by relative stability before declining from the late 50s (see Fig. 6) (Grydeland et al., 2013). Cognitive functioning, as measured by intraindividual variability in performance during a speeded task, correlated with both T1/T2-weighted myelin mapping and MD, indicating that higher degree of intracortical myelin is associated with greater performance stability throughout the lifespan.

However, the exact break point where maturation ends and aging or disease processes begin may not easily be identified for many imaging parameters. As for white matter volume, similar ambiguous findings may be obtained. As seen from the above, FA starts declining while volumetric increase is still present in the aging brain (Westlye et al., 2010). What underlying cellular events cause these different white matter imaging parameters to diverge, and what does it mean? As reviewed by Concha, it has been shown that influences such as neurodegeneration in crossing fibers, or highly organized glial cells in scar tissue in the brain, may yield seemingly paradoxical increases in diffusion anisotropy (Concha, 2014).

**Fig. 8:**
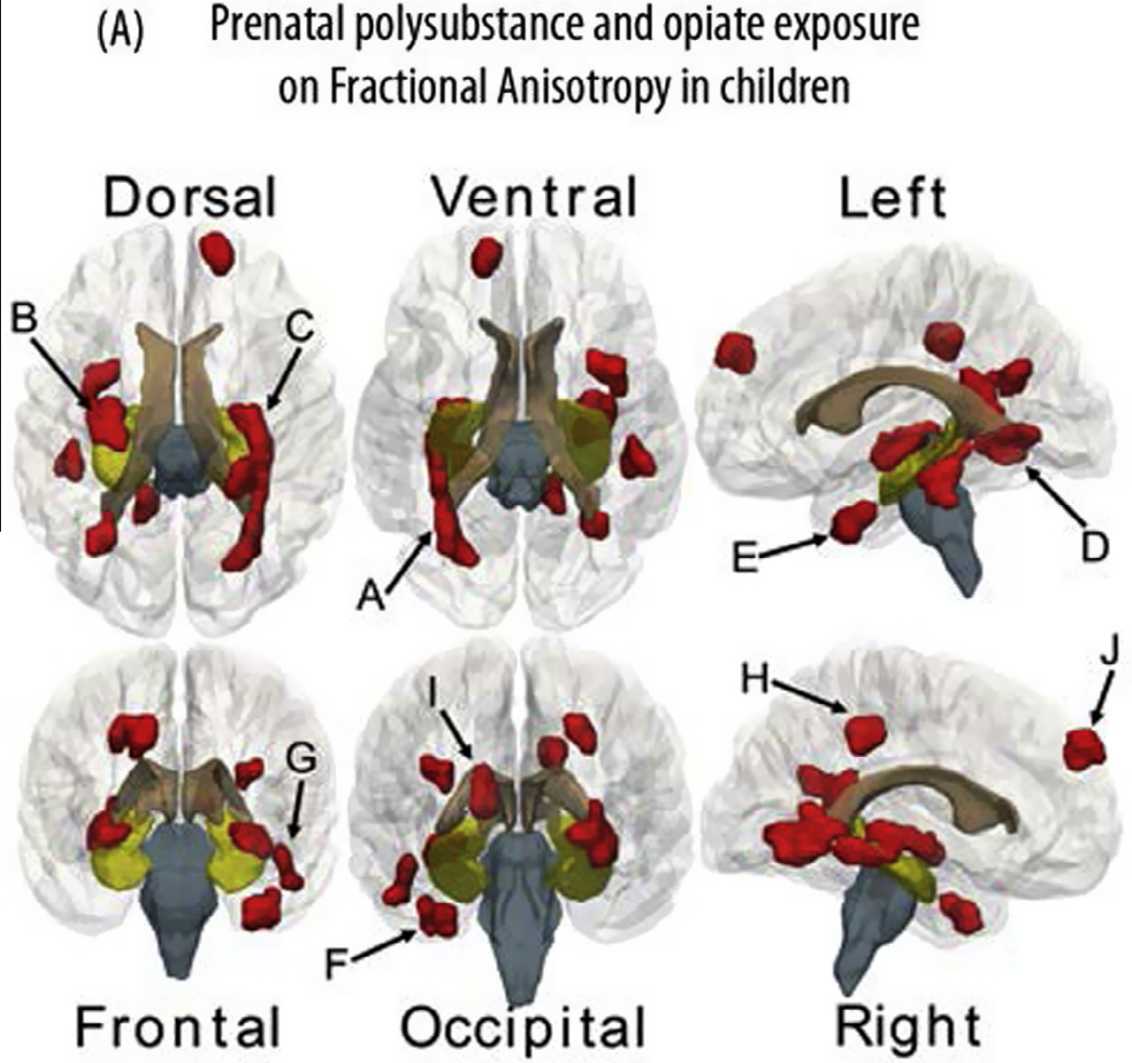
Areas of group differences in white matter microstructural properties between children prenatally exposed to opioids and other drugs versus controls. Clusters of voxels (⩾100) with significant (*P* < .05) group differences in fractional anisotropy (FA) are shown. For all clusters, FA was lower in the prenatally substance-exposed children. The depicted clusters are based on Walhovd et al. (2010).

In select cases of pathology as discussed above, we have the rare cases where human histological confirmation may enlighten the interpretation of imaging findings. Unfortunately, we are a relatively long way from achieving the same kind of understanding in normal lifespan changes. Brain banks for previously investigated participants may add information to some extent. It would be extremely helpful to achieve a greater understanding of the neurobiological underpinnings of observed imaging parameters if one prior to removing brains and treating them for slicing and select histological analyses, could scan them also at commonly used field strengths. Especially if one were to obtain such data for persons at different ages who passed away from other causes than brain disease or injury, that would allow valuable comparative analyses of imaging and histological data. Change itself must however be sampled *in vivo*, and preferably over prolonged time periods. Hence, we must rely on integration of cellular and multimodal imaging findings, animal and human data, to better utilize our knowledge to understand white matter changes in the brain across also the human lifespan, and their relation to optimal and non-optimal cognitive function. This is also increasingly important as imaging markers of white matter are more and more often studied and utilized for understanding the relation of human brain and cognitive changes and identifying negative and positive effects of various influences on development and aging (see Fig. 8A for examples).

## Conclusion

Utilizing animal models to dissect the neuroanatomical underpinning of the signals measured in a single voxel will provide valuable information. But the difference between the human and the rodent white matter needs to be accounted for. Thus collaboration between cellular neuroscience, neuroanatomy, neuropathology and macroscopic imaging is needed to better understand human imaging studies and thereby the human brain.

## References

[b0005] Alexander D.C., Hubbard P.L., Hall M.G., Moore E.A., Ptito M., Parker G.J., Dyrby T.B. (2010). Orientationally invariant indices of axon diameter and density from diffusion MRI. Neuroimage.

[b0010] Almeida R.G., Lyons D.A. (2014). On the resemblance of synapse formation and CNS myelination. Neuroscience.

[b0015] Amlien I., Fjell A.M. (2014). Diffusion tensor imaging of white matter degeneration in Alzheimer’s disease and mild cognitive impairment. Neuroscience.

[b0020] Assaf Y., Blumenfeld-Katzir T., Yovel Y., Basser P.J. (2008). Axcaliber: a method for measuring axon diameter distribution from diffusion MRI. Magn Reson Med.

[b0025] Basser P.J., Mattiello J., LeBihan D. (1994). Estimation of the effective self-diffusion tensor from the NMR spin echo. J Magn Reson B.

[b0030] Beaulieu C., Johansen-Berg H., Behrens T.E.J. (2009). Diffusion MRI: from quantitative measurement to in-vivo neuroanatomy.

[b0035] Behrens T.E., Johansen-Berg H., Jbabdi S., Rushworth M.F., Woolrich M.W. (2007). Probabilistic diffusion tractography with multiple fibre orientations: what can we gain?. Neuroimage.

[b0040] Bennett I.J., Madden D.J. (2014). Disconnected aging: cerebral white matter integrity and age-related differences in cognition. Neuroscience.

[b0045] Blumenfeld-Katzir T., Pasternak O., Dagan M., Assaf Y. (2011). Diffusion MRI of structural brain plasticity induced by a learning and memory task. PLoS One.

[b0050] Brown T.T., Jernigan T.L. (2012). Brain development during the preschool years. Neuropsychol Rev.

[b0055] Brown T.T., Kuperman J.M., Chung Y., Erhart M., McCabe C., Hagler D.J., Venkatraman V.K., Akshoomoff N., Amaral D.G., Bloss C.S., Casey B.J., Chang L., Ernst T.M., Frazier J.A., Gruen J.R., Kaufmann W.E., Kenet T., Kennedy D.N., Murray S.S., Sowell E.R., Jernigan T.L., Dale A.M. (2012). Neuroanatomical assessment of biological maturity. Curr Biol.

[b0060] Chau W.K., So K.F., Tay D., Dockery P. (2000). A morphometric study of optic axons regenerated in a sciatic nerve graft of adult rats. Restor Neurol Neurosci.

[b0065] Chen J.J., Rosas H.D., Salat D.H. (2013). The relationship between cortical blood flow and sub-cortical white-matter health across the adult age span. PLoS One.

[b0070] Chomiak T., Hu B. (2009). What is the optimal value of the g-ratio for myelinated fibers in the rat CNS? A theoretical approach. PloS One.

[b0075] Concha L. (2014). A macroscopic view of microstructure: using diffusion weighted images to infer damage, repair and plasticity of white matter. Neuroscience.

[b0080] Dawson M.R., Polito A., Levine J.M., Reynolds R. (2003). NG2-expressing glial progenitor cells: an abundant and widespread population of cycling cells in the adult rat CNS. Mol Cell Neurosci.

[b0085] Dekaban A.S. (1978). Changes in brain weights during the span of human life: relation of brain weights to body heights and body weights. Ann Neurol.

[b0090] Demerens C., Stankoff B., Logak M., Anglade P., Allinquant B., Couraud F., Zalc B., Lubetzki C. (1996). Induction of myelination in the central nervous system by electrical activity. Proc. Natl. Acad. Sci..

[b0095] Dubois J., Dehaene-Lambertz G., Kulikova S., Poupon C., Huppi P.S., Hertz-Pannier L. (2014). The early development of brain white matter: a review of imaging studies in fetuses, newborns and infants. Neuroscience.

[b0100] Engvig A., Fjell A.M., Westlye L.T., Moberget T., Sundseth O., Larsen V.A., Walhovd K.B. (2012). Memory training impacts short-term changes in aging white matter: a longitudinal diffusion tensor imaging study. Hum Brain Mapp.

[b0105] Fischl B., Salat D.H., Busa E., Albert M., Dieterich M., Haselgrove C., van der Kouwe A., Killiany R., Kennedy D., Klaveness S., Montillo A., Makris N., Rosen B., Dale A.M. (2002). Whole brain segmentation: automated labeling of neuroanatomical structures in the human brain. Neuron.

[b0110] Gibson E.M., Purger D., Mount C.W., Goldstein A.K., Lin G.L., Wood L.S., Inema I., Miller S.E., Bieri G., Zuchero J.B., Barres B.A., Woo P.J., Vogel H., Monje M. (2014). Neuronal activity promotes oligodendrogenesis and adaptive myelination in the mammalian brain. Science.

[b0115] Gilmore J.H., Shi F., Woolson S.L., Knickmeyer R.C., Short S.J., Lin W., Zhu H., Hamer R.M., Styner M., Shen D. (2012). Longitudinal development of cortical and subcortical gray matter from birth to 2 years. Cereb Cortex.

[b0120] Glasser M.F., Van Essen D.C. (2011). Mapping human cortical areas in vivo based on myelin content as revealed by T1- and T2-weighted MRI. J Neurosci.

[b0125] Grydeland H., Walhovd K.B., Tamnes C.K., Westlye L.T., Fjell A.M. (2013). Intracortical myelin links with performance variability across the human lifespan: results from T1- and T2-weighted MRI myelin mapping and diffusion tensor imaging. J Neurosci.

[b0130] Herculano-Houzel S. (2011). Not all brains are made the same: new views on brain scaling in evolution. Brain Behav Evol.

[b0135] Herculano-Houzel S. (2014). The glia/neuron ratio: how it varies uniformly across brain structures and species and what that means for brain physiology and evolution. Glia.

[b0140] Huttenlocher P.R., de Courten C., Garey L.J., Van der Loos H. (1982). Synaptogenesis in human visual cortex–evidence for synapse elimination during normal development. Neurosci Lett.

[b0145] Jog A., Roy S., Carass A., Prince J.L. (2013). Pulse sequence based multi-acquisition MR intensity normalization. Proc. SPIE.

[b0150] Johansen-Berg H. (2010). Behavioural relevance of variation in white matter microstructure. Curr Opin Neurol.

[b0155] Johnson R.T., Yeatman J.D., Wandell B.A., Buonocore M.H., Amaral D.G., Nordahl C.W. (2013). Diffusion properties of major white matter tracts in young, typically developing children. Neuroimage.

[b0160] Jones D.K., Simmons A., Williams S.C., Horsfield M.A. (1999). Non-invasive assessment of axonal fiber connectivity in the human brain via diffusion tensor MRI. Magn Reson Med.

[b0165] Kakita A., Zerlin M., Takahashi H., Goldman J.E. (2003). Some glial progenitors in the neonatal subventricular zone migrate through the corpus callosum to the contralateral cerebral hemisphere. J Comp Neurol.

[b0170] Káradóttir R., Cavelier P., Bergersen L.H., Attwell D. (2005). NMDA receptors are expressed in oligodendrocytes and activated in ischaemia. Nature.

[b0175] Káradóttir R., Hamilton N.B., Bakiri Y., Attwell D. (2008). Spiking and nonspiking classes of oligodendrocyte precursor glia in CNS white matter. Nat Neurosci.

[b0180] Kukley M., Capetillo-Zarate E., Dietrich D. (2007). Vesicular glutamate release from axons in white matter. Nat Neurosci.

[b0185] Le Bihan D., Breton E., Lallemand D., Grenier P., Cabanis E., Laval-Jeantet M. (1986). MR imaging of intravoxel incoherent motions: application to diffusion and perfusion in neurologic disorders. Radiology.

[b0190] Lundgaard I., Luzhynskaya A., Stockley J.H., Wang Z., Evans K.A., Swire M., Volbracht K., Gautier H.O.B., Franklin R.J.M., ffrench-Constant C., Attwell D., Káradóttir R.T. (2013). Neuregulin and BDNF induce a switch to NMDA receptor-dependent myelination by oligodendrocytes. PLoS Biol.

[b0195] Lundgaard I., Osório M.J., Kress B.T., Sanggaard S., Nedergaard M. (2014). White matter astrocytes in health and disease. Neuroscience.

[b0200] Makedonov I., Black S.E., Macintosh B.J. (2013). BOLD fMRI in the white matter as a marker of aging and small vessel disease. PLoS One.

[b0205] Marner L., Nyengaard J.R., Tang Y., Pakkenberg B. (2003). Marked loss of myelinated nerve fibers in the human brain with age. J Comp Neurol.

[b0210] Mitew S., Hay C.M., Peckham H., Xiao J., Koenning M., Emery B. (2014). Mechanisms regulating the development of oligodendrocytes and central nervous system myelin. Neuroscience.

[b0215] Mutsaerts H.J., Richard E., Heijtel D.F., van Osch M.J., Majoie C.B., Nederveen A.J. (2013). Gray matter contamination in arterial spin labeling white matter perfusion measurements in patients with dementia. NeuroImage Clinical.

[b0220] Oberheim N.A., Takano T., Han X., He W., Lin J.H.C., Wang F., Xu Q., Wyatt J.D., Pilcher W., Ojemann J.G. (2009). Uniquely hominid features of adult human astrocytes. J Neurosci.

[b0225] Østby Y., Tamnes C.K., Fjell A.M., Westlye L.T., Due-Tønnessen P., Walhovd K.B. (2009). Heterogeneity in subcortical brain development: a structural magnetic resonance imaging study of brain maturation from 8 to 30 years. J Neurosci.

[b0230] Pajevic S., Basser P.J., Fields R.D. (2014). Role of myelin plasticity in oscillations and synchrony of neuronal activity. Neuroscience.

[b0235] Parker G.J., Alexander D.C. (2005). Probabilistic anatomical connectivity derived from the microscopic persistent angular structure of cerebral tissue. Philos Trans R Soc Lond B Biol Sci.

[b0240] Paus T., Pesaresi M., French L. (2014). White matter as a transport system. Neuroscience.

[b0245] Petrella J.R., Provenzale J.M. (2000). MR perfusion imaging of the brain: techniques and applications. AJR Am J Roentgenol.

[b0250] Reuter M., Schmansky N.J., Rosas H.D., Fischl B. (2012). Within-subject template estimation for unbiased longitudinal image analysis. Neuroimage.

[b0255] Rosas H.D., Liu A.K., Hersch S., Glessner M., Ferrante R.J., Salat D.H., van der Kouwe A., Jenkins B.G., Dale A.M., Fischl B. (2002). Regional and progressive thinning of the cortical ribbon in Huntington’s disease. Neurology.

[b0260] Sadeghi N., Prastawa M., Fletcher P.T., Wolff J., Gilmore J.H., Gerig G. (2013). Regional characterization of longitudinal DT-MRI to study white matter maturation of the early developing brain. Neuroimage.

[b0265] Salat D.H. (2014). Imaging small vessel-associated white matter changes in aging. Neuroscience.

[b0270] Sampaio-Baptista C., Khrapitchev A.A., Foxley S., Schlagheck T., Scholz J., Jbabdi S., DeLuca G.C., Miller K.L., Taylor A., Thomas N., Kleim J., Sibson N.R., Bannerman D., Johansen-Berg H. (2013). Motor skill learning induces changes in white matter microstructure and myelination. J Neurosci.

[b0275] Scholz J., Klein M.C., Behrens T.E., Johansen-Berg H. (2009). Training induces changes in white-matter architecture. Nat Neurosci.

[b0280] Seidl A.H. (2014). Regulation of conduction time along axons. Neuroscience.

[b0285] Small R.K., Riddle P., Noble M. (1987). Evidence for migration of oligodendrocyte–type-2 astrocyte progenitor cells into the developing rat optic nerve. Nature.

[b0290] Snaidero N., Möbius W., Czopka T., Hekking L.H.P., Mathisen C., Verkleij D., Goebbels S., Edgar J., Merkler D., Lyons D.A., Nave K.-A., Simons M. (2014). Myelin membrane wrapping of CNS axons by PI(3,4,5)P3-dependent polarized growth at the inner tongue. Cell.

[b0295] Song S.K., Sun S.W., Ju W.K., Lin S.J., Cross A.H., Neufeld A.H. (2003). Diffusion tensor imaging detects and differentiates axon and myelin degeneration in mouse optic nerve after retinal ischemia. Neuroimage.

[b0300] Stevens B., Porta S., Haak L.L., Gallo V., Fields R.D. (2002). Adenosine: a neuron-glial transmitter promoting myelination in the CNS in response to action potentials. Neuron.

[b0305] Sturrock R.R. (1980). Myelination of the mouse corpus callosum. Neuropathol Appl Neurobiol.

[b0310] Takeuchi H., Sekiguchi A., Taki Y., Yokoyama S., Yomogida Y., Komuro N., Yamanouchi T., Suzuki S., Kawashima R. (2010). Training of working memory impacts structural connectivity. J Neurosci.

[b0315] Tamnes C.K., Ostby Y., Fjell A.M., Westlye L.T., Due-Tonnessen P., Walhovd K.B. (2010). Brain maturation in adolescence and young adulthood: regional age-related changes in cortical thickness and white matter volume and microstructure. Cereb Cortex.

[b0320] Taubert M., Draganski B., Anwander A., Muller K., Horstmann A., Villringer A., Ragert P. (2010). Dynamic properties of human brain structure: learning-related changes in cortical areas and associated fiber connections. J Neurosci.

[b0325] Tomassy G.S., Berger D.R., Chen H.-H., Kasthuri N., Hayworth K.J., Vercelli A., Seung H.S., Lichtman J.W., Arlotta P. (2014). Distinct profiles of myelin distribution along single axons of pyramidal neurons in the neocortex. Science.

[b0330] Treit S., Chen Z., Rasmussen C., Beaulieu C. (2014). White matter correlates of cognitive inhibition during development: a diffusion tensor imaging study. Neuroscience.

[b0335] van Oort E.S., van Cappellen van Walsum A.M., Norris D.G. (2013). An investigation into the functional and structural connectivity of the default mode network. Neuroimage.

[b0340] van Osch M.J., Teeuwisse W.M., van Walderveen M.A., Hendrikse J., Kies D.A., van Buchem M.A. (2009). Can arterial spin labeling detect white matter perfusion signal?. Magn Reson Med.

[b9005] Walhovd K.B., Westlye L.T., Moe V., Slinning K., Due-Tonnessen P., Bjornerud A., van der Kouwe A., Dale A.M., Fjell A.M. (2010). White matter characteristics and cognition in prenatally opiate- and polysubstance-exposed children: a diffusion tensor imaging study. AJNR Am J Neuroradiol.

[b0345] Walhovd K.B., Westlye L.T., Amlien I., Espeseth T., Reinvang I., Raz N., Agartz I., Salat D.H., Greve D.N., Fischl B., Dale A.M., Fjell A.M. (2011). Consistent neuroanatomical age-related volume differences across multiple samples. Neurobiol Aging.

[b0350] Wang S., Young K.M. (2014). White matter plasticity in adulthood. Neuroscience.

[b0355] Wedeen V.J., Hagmann P., Tseng W.Y., Reese T.G., Weisskoff R.M. (2005). Mapping complex tissue architecture with diffusion spectrum magnetic resonance imaging. Magn Reson Med.

[b0360] Westlye L.T., Walhovd K.B., Dale A.M., Bjornerud A., Due-Tonnessen P., Engvig A., Grydeland H., Tamnes C.K., Ostby Y., Fjell A.M. (2010). Life-span changes of the human brain White matter: diffusion tensor imaging (DTI) and volumetry. Cereb Cortex.

[b0365] Wheeler-Kingshott C.A., Cercignani M. (2009). About “axial” and “radial” diffusivities. Magn Reson Med.

[b0370] White N.S., Leergaard T.B., D’Arceuil H., Bjaalie J.G., Dale A.M. (2013). Probing tissue microstructure with restriction spectrum imaging: histological and theoretical validation. Hum Brain Mapp.

[b0375] Zatorre R., Fields R.D., Johansen-Berg H. (2012). Plasticity in gray and white: Neuroimaging changes in brain structure during learning. Nat Neurosci.

[b0380] Ziskin J.L., Nishiyama A., Rubio M., Fukaya M., Bergles D.E. (2007). Vesicular release of glutamate from unmyelinated axons in white matter. Nat Neurosci.

[b0385] Zito G., Luders E., Tomasevic L., Lupoi D., Toga A.W., Thompson P.M., Rossini P.M., Filippi M.M., Tecchio F. (2014). Inter-hemispheric functional connectivity changes with corpus callosum morphology in multiple sclerosis. Neuroscience.

